# Current treatment strategies for seasonal allergic rhinitis: where are we heading?

**DOI:** 10.1186/s12948-022-00176-x

**Published:** 2022-08-10

**Authors:** Erminia Ridolo, Cristoforo Incorvaia, Francesco Pucciarini, Elena Makri, Giovanni Paoletti, Giorgio Walter Canonica

**Affiliations:** 1 Dept. Medicine and Surgery, University of Parma, Parma, Italy; 2 Niguarda Ca’ Granda Hospital, Milan, Italy; 3 Personalized Medicine, Asthma and Allergy, Humanitas Clinical and Research Hospital, IRCCS, Rozzano, MI Italy; 4 Department of Biomedical Sciences, Humanitas University, Pieve Emanuele, MI Italy

**Keywords:** Seasonal allergic rhinitis, Treatment strategy, Symptomatic drugs, Allergen immunotherapy, Personalized medicine

## Abstract

**Introduction:**

Allergic rhinitis (AR) is very commonly caused by pollens. The symptoms of AR consist of sneezing, nasal congestion, rhinorrhea, nasal itching and airflow obstruction. The diagnosis has long been based on clinical history, skin prick tests and in vitro measurement of specific IgE, but the innovative approach of precision medicine has made diagnostic tools of much greater accuracy available.

**Areas covered:**

This review covers the advances in the treatment of seasonal AR concerning the drugs to be used according to the grade of disease and the characteristics of the patients, and the role of allergen immunotherapy (AIT), which is the only treatment capable of acting, in addition to the symptoms, on the cause of AR and therefore to modify its natural history.

**Expert opinion:**

Drug treatment of AR include a large number of agents, the choice of which depends on the severity of the disease. AIT has high evidence of efficacy demonstrated by meta-analyses, and further improvement is currently apparent, as for diagnosis, applying the means of precision medicine. However, when AIT is performed in current practice, without the strict rules of controlled trials, long-term low adherence is a major problem to be solved.

## Introduction

Allergic rhinitis (AR) is very commonly caused by pollens. The major culprit, due to its presence in all temperate zones, is grass pollen. A recent study on a large birth cohort found that the prevalence of sensitization to Phleum pratense was 9.7% at 4 years, 28.4% at 8 years and 37.1% at 16 years [1]. Further causes of seasonal AR are other pollens belonging to different plant families such as Asteracee, Urticacee, Cupressacee, Betulacee, and Oleacee, with variable importance in the different areas of the world. Symptoms of AR comprise rhinorrea, nasal congestion, sneezing, nasal itching and airflow obstruction, although other related symptoms can also occur [2]. It has long been known that AR has its peak of prevalence in the second to fourth decades of life, and then gradually declines [3]. AR is also a known risk factor for subsequent development of asthma [4, 5].

Several clinical variants of AR have been classified in the past, but the first edition of the document “Allergic Rhinitis and its Impact on Ashtma” (ARIA) reduced them to only the intermittent and persistent forms [6]. As far as diagnosis is concerned, for a long time the only diagnostic tool available was the analysis of the patient’s clinical history. Today we would tend to think that in patients with symptoms during the time of the year corresponding to a precise pollination of a plant, no further investigation is needed. However, most of patients are polysensitized and even in patients with a monosensitization (rare in real life) the critical period may be shared by different pollens. Therefore an allergy test has to be done possibly in any patients, to confirm the diagnosis.

**Fig. 1:**
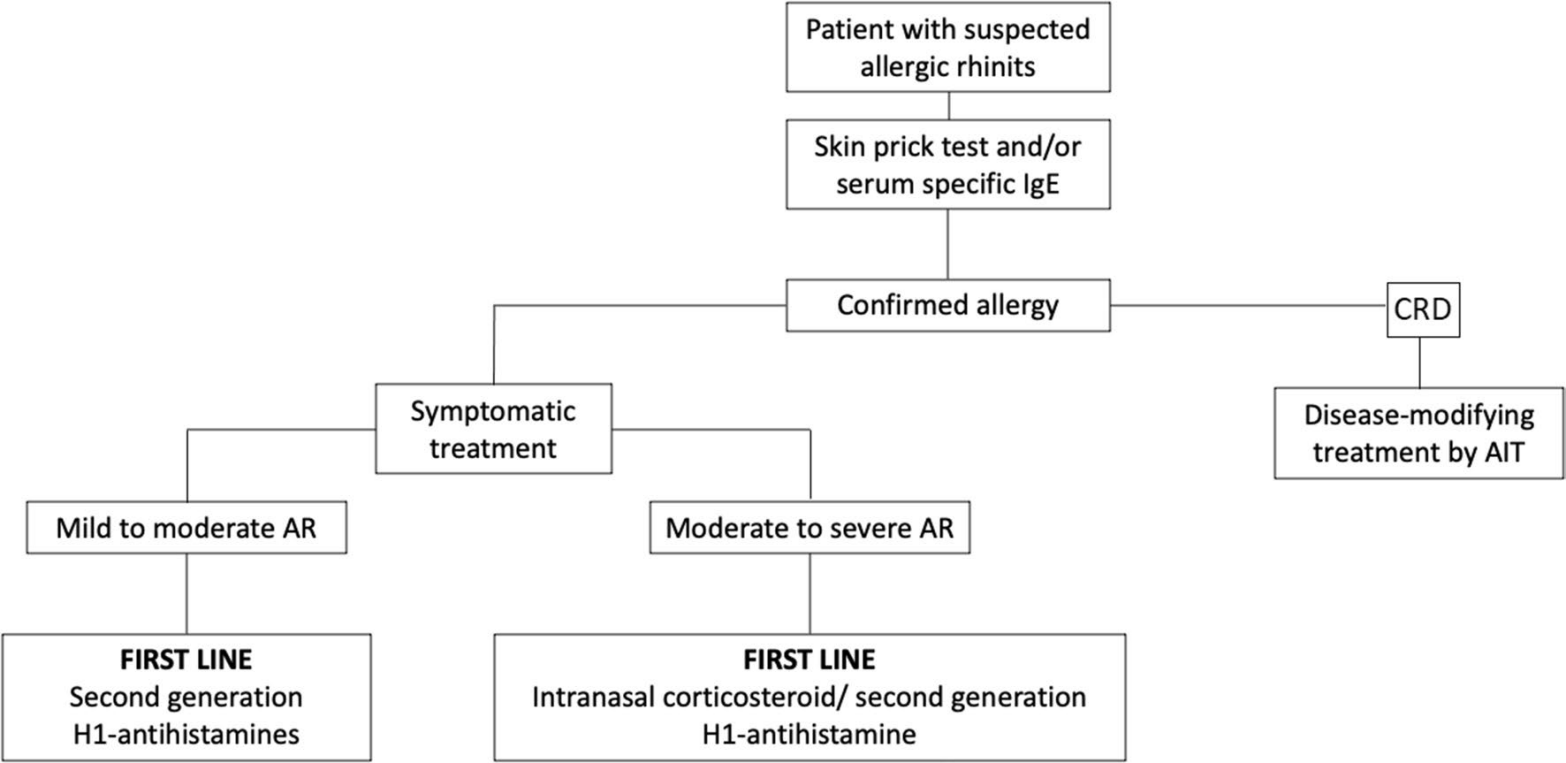
Algorithm of treatment strategies for allergic rhinitis The figure shows how the treatment can be only symptomatic or even disease-modifying through a tailor treatment on the patient, once obtained the etiological diagnosis of seasonal rhinitis

Precision medicine, which has tools such as component resolved diagnosis (CRD), able to detect specific single-allergen molecules, allow to discern the causative allergens from the simply cross-reactive ones, while defining the patient's treatable traits addressing genetic and phenotypic features, and also omics, to predict the patient's response to the therapy [7] (Fig. 1).

## Kind of treatment for AR

The treatment approach for AR may be based on drugs aimed at repealing or reducing allergic symptoms or on allergen immunotherapy (AIT), which is instead designed to modify the immunological response to the causative allergen inducing its tolerance. However, these two treatments have synergistic effect on patients and are not alternatives. Symptomatic treatments for AR are commonly dispensed as over the counter drug by pharmacists. In 2015, a survey performed in Italy showed that 87% of pharmacists, compared to 49% of general practitioners, were unaware of the ARIA guidelines [8]. Thus, the most recent ARIA document stated that, as community pharmacists are for AR patients the most approachable healthcare professionals, “the development of an integrated pathway in which the pharmacist is a member of the interdisciplinary team can affect the quality of both the individual healthcare services and the patient’s healthcare plan” [9].

Since the estimated prevalence of AR ranges from 20 to 30% of the population in both Europe and United States [10], it goes without saying that drug therapy, commonly prescribed by general practitioners, is much more used than AIT, which is prescribed by specialists. Various guidelines are available to address opportunities for quality improvement for clinicians in different settings, in order to optimize the management and care of AR patients and to promote diagnosis's and therapy's effectiveness [3, 11].

## Current drug treatment

The International Consensus Statement on allergic rhinitis dedicated a section to the evidence of efficacy for the different drugs used to treat AR, including intranasal and oral antihistamines, intranasal, oral, and injectable corticosteroids, oral and intranasal decongestant, leukotriene receptor antagonist, oral cromolyn, intranasal anticholinergics and biologics (omalizumab), as measured through the Aggregate Grade of Evidence (AGE) [12]. Table 1 shows the respective AGE values to any drug and, when available, its different pharmaceutical preparations, based on the evaluation of studies that received a level based on the Oxford LOE, and the subsequent recommendation level.

**Table 1:** Efficacy of drugs to treat AR and recommendation level, according to Aggregate Grade of Evidence (AGE) from International Consensus Statement on Allergy and Rhinology: Allergic Rhinitis [12]

Type of drug and pharmaceutical preparations	AGE	Number of listed studies	Recommendation level
Oral H_1_ antihistamines	A	Level 1a: 21 studies	Strong recommendation
Intranasal antihistamines	A	Level 1b: 43 studies Level 2b: 1 study	Recommendation
Intranasal corticosteroids (INCS)	A	Level 1a: 15 studies Level 1b: 33 studies Level 2a: 3 studies Level 2b: 1 study Level 5: 1 study	Strong recommendation
Combination: INCS and intranasal antihistamine	A	Level 1b: 9 studies Level 2b: 1 study Level 2c: 2 studies	Strong recommendation
Oral corticosteroids	B	Level 1b: 5 studies Level 2b: 1 study Level 4: 3 studies	Recommendation against
Injectable corticosteroids	B	Level 1b: 3 studies Level 2b: 3 studies Level 4: 7 studies	Recommendation against
Oral decongestants	B	Level 1a: 2 studies Level 1b: 3 studies Level 3b: 2 studies Level 4: 2 studies	Option for pseudoephedrine for short term treatment Recommend against phenlylephrine
Intranasal decongestants	B	Level 1b: 3 studies Level 2b: 1 study	Option
Leukotriene receptor antagonists	A	Level 1a: 6 studies Level 1b: 17 studies Level 2a: 2 studies Level 2b: 3 studies Level 4: 3 studies	Recommendation against
Cromolyn (DSCG)	A	Level 1b: 13 studies Level 2b: 9 studies	Option
Intranasal anticholinergics (IPB)	B	Level 1b: 9 studies Level 2b: 5 studies	Option
Biologics (omalizumab)	A	Level 1a: 1 study Level 1b: 5 studies	No indication

Aggregate grade of evidence (AGE)	
Grade	Research quality
A	Well-designed RCTs
B	RCTs with minor limitations; overwhelming consistent evidence from observational studies
C	Observational studies (case control and cohort design)
D	Expert opinion; Case reports; Reasoning from first principles

Another interesting approach is based on optimizing the treatment and disease management by detecting patients with severe AR. A large multicenter French study on seasonal AR assessed the patients with a 17 items questionnaire (Allergic Rhinitis Physician Score (ARPhyS) and the commonly used total symptom score (TSS-17). Patients were stratified according to AR severity and categorized into “mild”, “moderate”, and “severe”, through five different methods. The ARPhyS scale proved to be the best in discriminating the patients' AR severity, with reported cut-offs at the score of 8 to 9 for mild to moderate AR, and of 11 to 12 for moderate to severe AR. TSS-17 also had excellent score reliability (0.864), while Cronbach’s α coefficient was considered only acceptable (0.626). The authors then concluded that the ARPhyS scale could be a useful tool in the general practitioner daily practice, to identify those patients with severe AR in need of the specialist’s interventions [13].

## Personalized medicine

The concept of personalized medicine, which is based on treating the patient and not just the disease, as previously done for centuries, is currently deeply changing the practice of medicine and particularly the therapy [14].

### Personalized medicine for drug treatment

The first study using the personalized medicine approach analyzed the outcome of different drugs to treat AR in elderly patients. The results highlighted important matters: first and second generation antihistamines demonstrated a high incidence of adverse effects and drug to drug interactions; oral decongestants were risky when a variety of comorbidities common in older people were present. Leukotriene receptor antagonists were as effective as antihistamines, but less effective than intranasal corticosteroids, which showed the greatest safety and efficacy profile. The authors concluded that the approach for the diagnosis and treatment of AR in elderly patients should be tailored to their specific age-related factors [15]. Further studies evaluated particular applications of personalized medicine. They included: personalized pollen-related symptom-forecast information services for european patients with AR [16]; serum cytokine profiling as an indicator for personalized treatment of allergy [17]; validation of a new molecular multiplex IgE assay for the diagnosis and phenotipization of pollen allergy in the Mediterranean area [18]; implementation of digital technologies, such as online platforms for both patients and physicians, to improve the management of respiratory allergic diseases [19]. It is conceivable that in the years to come the number of studies dedicated to this important topic will increase more and more.

### Personalized medicine for allergen immunotherapy

The literature on personalized medicine applications for allergen immunotherapy (AIT) is significantly more abundant. Actually, AIT is perfectly suited to the three needs to be met in personalized medicine: identification of the disease's molecular mechanism, availability of a diagnostic tool able to recognize such mechanism, and a treatment capable of blocking the mechanism itself [20]. Indeed, also before the introduction of personalized medicine, a number of meta-analyses proved the effectiveness of both subcutaneous and sublingual immunotherapy in patients with seasonal allergic rhinitis [21–24]. In two of them, the outcome of AIT was compared to pharmacotherapy. The first meta-analysis demonstrated that the relative clinical impact of AIT was higher than those of mometasone and montelukast [22]; in the second one the relative clinical impact of AIT was greater than second-generation antihistamines and leukotriene receptor antagonists, while comparable to nasal corticosteroids [24]. As for the growing number of studies related to personalized medicine, those of highest interest will be discussed here. Variable personalized methods were used. In the first study, a deep analysis of allergenome in patients allergic to Japanese cedar (the most common seasonal allergy in Japan) was performed using immunoblotting analysis combined with two-dimensional electrophoresis. A number of novel IgE-reactive allergen molecules, including a serine protease, an aspartic protease, a lipid transfer protein, chitinase, as well as some novel IgE-reactive molecules, were found to have potential capacity to improve the diagnostic precision and consequently the effectiveness of AIT [25]. A subsequent study, based on one of the already known mechanisms that cause Th2 dominance, the inhibition of naive T cells differentiation in Th1 cells, tested the AIT ability to produce a shift towards Th1 dominance, presuming an alteration in interferon type I signaling caused by the therapy. Thus, the authors analyzed allergen and diluent challenged CD4^+^ T cell, from patients at different time points and from a healthy control group. The first results showed complex changes subsequent to AIT, consistent with the authors’ hypothesis of an interferon signaling pathway involving a different number of genes. However, the authors claimed the need for the result's validation in a larger group of patients [26]. More recently, a study in patients allergic to Artemisia pollen analyzed the clinical responses before and after 1 year of AIT, to identify responders to treatment by measure of specific IgE and IgG4 levels using ImmunoCAP and ELISA. Stepwise regression analysis was used to define which rhinitis-relevant parameters explained the variability in the outcome of the therapy. Only the responders had high levels of Artemisia specific IgE and IgG4. Analyzing the association to allergy and protein fold changes, four candidate biomarkers emerged as candidate biomarkers to predict AIT efficacy, while further ELISA proved that only the leukotriene A_4_ hydrolase was consistent with the proteomics data, showing a significant increase in responders after 1 year of AIT, while non-responders showed no significant changes. Based on these results, the authors suggested that serum LTA_4_H could be used as a valid biomarker for early prediction of effective AIT [27]. A European Academy of Allergy and Clinical Immunology (EAACI) position paper addressed the potential role of mobile health technologies and applications, including personal devices, such as smartphones and tablet, to sustain and improve health-related services, patients’ self-management, surveillance, and disease management after the first diagnosis of AR, with the aim to optimize the patient’s therapy. A team of experts was then created, to define the current state of the art and the forthcoming potential of mobile health technologies in the field of allergology. Endorsing the "Be He@lthy, Be Mobile" WHO initiative, the quality, usability, efficiency, advantages, limitations, and eventual risks of these solutions used in allergic patients were matters of debate. Also, the regulatory context related to the "General Data Protection Regulation" and Medical Directives of the European Community was implemented. The observations of healthcare workers and allergic patients underlined the demand of in-depth investigation to ensure an effective design of mHealth technologies as supplementary tools to improve the quality of care. In the context of the personalized medicine, these tools might be useful in changing the perspective from a clinician to a patient-centered care. The impact of mobile technologies and the associated big data sets were defined, in different areas of allergology, ranging from allergic rhinitis to asthma, from dermatological diseases to food allergies, insect venom and drug allergy, and also in the field of immunotherapy [28]. Mikus et al. produced an allergome-wide microarray, including 731 allergens and over 172,000 overlapping 16-mer peptides. IgE, IgG_4_, and IgG allergen recognition was analyzed in samples of serum collected from AR subjects undergoing AIT for pollen allergy. The study showed wide-ranging induction of Phl p 1 and Bet v 1-specific humoral immunity in subjects after 3 years of AIT for grass and birch allergy. Different profiles based on the different epitopes detected were found mainly after 1 year of AIT, indicating that the leading allergen-specific clones persists as a fundamental supplier to humoral immunity even after their preliminary creation during the first phases of the therapy. Pattern of different allergen isoforms specific for different subjects were detected as group cross-reactivities, which might imply different grades of protection against various allergen sources. The authors suggested that the deconvolution of the epitopes could be an important topic for future attempts to evaluate the results of AIT in a personalized practice [29]. The most recent study to date assessed the usableness and impact of an algorithm for decisional support system (@IT2020-CDSS) in seasonal AR caused by either pollens or Alternaria. The diagnostic methods included clinical history, in vivo and in vitro test, in particular component resolved diagnosis (CRD), real-time digital symptom recording, and eDiary on prescription of AIT by the doctor. Following an educational preparation on the @IT2020-CDSS algorithm, a group of doctors (18 allergy specialists, and 28 general practitioners (GP), expressed a hypothetical AIT prescription in about ten different index cases. The association of eDiary and CRD improved the AIT decisions from the group of allergist as for the GPs. Based on history and proved sensitization to whole extracts, AIT prescription for sensitization to pollen or mold was heterogeneous, but the doctors reached a consensus in proposing integration of CRD and eDiary’s informations. The results suggest a potential usefulness of such approach and warrant further investigation [30].

## The unsolved problem of poor adherence to medical treatments

Adherence to medical therapy, as the degree to which a subject correctly follows medical advice [31], is of crucial importance in the clinical success of any treatment. A document from WHO stated that low adherence to therapy for chronic illnesses is a universal problem of outstanding relevance. In developed countries the adherence to long-standing therapies for chronic diseases is estimated to be 50%, even lower in developing countries. The burden of scarce adherence increases worldwide equally to the impact of chronic diseases, causing poor health outcomes and severely affecting the effectiveness of therapies, establishing an essential issue in population health, both in regard to quality of life as for the increased health care costs. Investments in secondary prevention of adverse health outcomes and primary prevention of risk factors would therefore have positives returns by clinical decisions aimed to increase adherence to treatments. As to possible solutions, the document stated that patients need to be not condemned but sustained, highlighting that despite indications of the contrary, there still be the trend to point on patient-related aspects as the main cause of poor adherence, while health providers and health system-related factors are important as well. Adherence is an active process in need of a long-term follow up, that requires patient-tailored interventions, while keeping in mind that there is no unique strategy, or group of strategies, that has proved to be successful for all situations, conditions and, in the end, patient. Thus, actions targeting adherence has to be tailored to the particular disease-related needs of the patient [32]. This problem also concerns AIT. In a systematic review including 9998 patients from 81 controlled trials on sublingual immunotherapy, the dropout rates appear not to be a relevant problem, as shown by an overall dropout rate of 14% [33]. However, it is known that randomized, placebo-controlled trials are based on rigid rules and established doctor-patient contact, while observational studies, and even more for current clinical practice, have a significantly lower outcome. [34]. In fact, an analysis based on the official data in continuation of current practice sublingual immunotherapy provided by the manufacturers of various products in 2010 showed an alarming rate of discontinuation, with only 10% of patients continuing the treatment at 3 years after the prescription. The authors defined the phenomenon as uniform and consistent, and suggested that its causes needed to be investigated urgently, because adherence is of primary relevance for the efficacy of SLIT [35]. Even worse results were obtained in a retrospective study published in 2013 from a community pharmacy database from The Netherlands, comprised of data from 6486 patients who started immunotherapy between 1994 and 2009. 2796 patients received subcutaneous immunotherapy and 3690 received sublingual immunotherapy. Globally, the minimally required duration of AIT [3 years] was achieved only by 18% of the subjects. Sublingual treatment had the worst result (7%), but also subcutaneous treatment resulted in bad compliance (23%). Prescribers proved to be independent predictors of early discontinuation, as patients of allergologists and other medical specialists showed shorter persistence than those of general practitioners; other predictors of discontinuation were younger age, lower socioeconomic status and single-allergen therapy [36]. However, the results of these kind of studies are uneven. For example, in a retrospective cohort analysis from a German longitudinal prescription database, the adherence rate after 2 years of AIT with tree pollen and grass allergoid was 60.1% for subcutaneous immunotherapy, while only 29.5% for the sublingual route. Children showed higher adherence than teenagers or adults. A large number of studies on the issue of adherence are available, but the basic concept is that adherence to AIT for inhalants is insufficient for the injection route and awfully insufficient for the sublingual route [37]. The problem must be addressed with commitment. Pitsios and Dietis have dealt with it by identifying and analyzing a number of interventions, including educational sessions. Before the start of AIT, is mandatory to schedule medical evaluations every 90 days, apart from the other visits; is important to keep in touch with the patients in case of pre-seasonal AIT treatment to remind the planned appointments, eventually with the help of a secretary; is also important to explain to the patients the different therapeutic options and try to obtain concordance (mainly in children), showing sincere interest on their opinions and worries, asking questions as: “how do you feel about the evidence that the road ahead you is much longer than the one already traveled since your last visit?”, mentioning the reach of time milestones and reminding them that the evidence of improvement tend to appear slow but will last for a long time after the end of AIT [38].

## Conclusion

The treatment of seasonal AR**,** even if both drug treatment and AIT have been used successfully for a very long time, has still room for improvement. The progress ensured by the innovative precision medicine has already brought enhancements in diagnosis, pharmacological therapy as well as AT, but it is apparent that the road ahead is still longer than the one traveled so far. In particular, the serious problem of poor adherence must be tackled with the utmost commitment, to avoid that an AIT treatment duration shorter than the recommended one of three consecutive years nullifies its effectiveness.

## Expert opinion

Seasonal AR has been known for centuries, and in 1911, when effective drugs were not available for its treatment, an innovative therapy called desensitization was proposed in the UK, which consisted of initially administering low and then progressively increasing doses of an extract of the grass Phleum pratense, to induce tolerance to the allergen [39]. Today that treatment is known as allergen immunotherapy, which is the only treatment capable of modifying the natural history of allergic conditions [40]. In the meantime, drug therapy has progressively been enriched with numerous agents suitable for treating the different forms and severities of AR. According to a recent review, there is a wide range of existing treatment options for AR, reflecting the variable disease severity and duration. Currently, newer generation antihistamines should be the first-line therapy for mild to moderate AR, while intranasal corticosteroids (INCS) are considered the mainstay treatment in case of moderate to severe AR. In patients with symptoms not controlled by INCS, their combination with additional drugs should be considered, presuming that cost is not a limiting factor [41]. As for the latest generation antihistamines, evidence of efficacy has been obtained for rupatadine by a systematic review of randomized placebo-controlled trials [42], and for bilastine through a multidisciplinary Real-World experience [43]. Concerning the combinations of intranasal formulations, to the first of them, consisting of azelastine and fluticasone [44], it has recently been added olopatadine hydrochloride and mometasone furoate, which demonstrated its efficacy for seasonal and perennial AR in a systematic review and meta-analysis [45].

As for any drug, side effects are possible. The greatest risk is borne by systemic corticosteroids, which must be reserved for the most serious cases. A recent consensus document addressed the efficacy of systemic steroids in the treatment of upper airway diseases as well as highlighting the possible harms of this therapy, providing recommendations for their use. Although with less frequency and severity than systemic corticosteroids, other drugs are also affected by side effects [46]. A recent review on AR management stated that a range of treatment options reflecting the varying disease length and severity is available. Between them, newer generation antihistamines for mild to moderate AR, and intranasal corticosteroids for more severe AR should be the mainstay treatment, even a combination of the two being feasible in the most demanding cases if its cost does not act as a limiting factor. The authors also took into consideration AIT, accomplishing that SCIT still is the option with the most possible targeted allergens, but the potential risk of severe systemic reactions demands weekly scheduled appointments for the administration in the first four to six months of therapy. SLIT has the advantage of being self-administered and is known for a lower risk for systemic reactions. For both treatments, a treatment plan dealing with AR severity and patient preferences is a mandatory standard care [47]. The future of seasonal allergy therapy will greatly benefit from personalized medicine. In a recent comprehensive analysis, Breiteneder et al. examined the modern health care system, which needs an active and individualized response to illnesses, starting from precision diagnosis and resulting in personalized therapy. Novel tools such as disease phenotyping and endotyping and the appliance of consistent biomarkers will become fundamental in the modern approach to allergic patients [48]. In the end, it is our opinion that, regardless the level of intervention, two major problems need to be addressed very seriously: the low adherence to treatments and, less considered but equally relevant, the poor technique for self-administering inhaled drugs, given that large numbers of patients do not use an adequate technique capable of allowing the drug to reach the site of action at the nasal level [12].

## Data Availability

Not applicable.
